# Hepatitis E Virus Infection: Circulation, Molecular Epidemiology, and Impact on Global Health

**DOI:** 10.3390/pathogens9100856

**Published:** 2020-10-20

**Authors:** Srinivas Reddy Pallerla, Dominik Harms, Reimar Johne, Daniel Todt, Eike Steinmann, Mathias Schemmerer, Jürgen J. Wenzel, Jörg Hofmann, James Wai Kuo Shih, Heiner Wedemeyer, C.-Thomas Bock, Thirumalaisamy P. Velavan

**Affiliations:** 1 Institute of Tropical Medicine, University of Tübingen, 72074 Tübingen, Germany; srinivas-reddy.pallerla@uni-tuebingen.de (S.R.P.); velavan@medizin.uni-tuebingen.de (T.P.V.); 2 Vietnamese-German Center for Medical Research (VG-CARE), Hanoi 100000, Vietnam; 3 Division of Viral Gastroenteritis and Hepatitis Pathogens and Enteroviruses, Department of Infectious Diseases, Robert Koch Institute, 13353 Berlin, Germany; HarmsD@rki.de; 4 Unit Viruses in Food, Department Biological Safety, German Federal Institute for Risk Assessment, 10589 Berlin, Germany; Reimar.Johne@bfr.bund.de; 5 Department of Molecular and Medical Virology, Ruhr University Bochum, 44801 Bochum, Germany; daniel.todt@ruhr-uni-bochum.de (D.T.); eike.steinmann@ruhr-uni-bochum.de (E.S.); 6 European Virus Bioinformatics Center (EVBC), 07743 Jena, Germany; 7 Institute of Clinical Microbiology and Hygiene, National Consultant Laboratory for HAV and HEV, University Medical Center Regensburg, 93053 Regensburg, Germany; Mathias.Schemmerer@klinik.uni-regensburg.de (M.S.); juergen.wenzel@klinik.uni-regensburg.de (J.J.W.); 8 Institute of Virology, Charité Universitätsmedizin Berlin, Labor Berlin-Charité-Vivantes GmbH, 13353 Berlin, Germany; Joerg.Hofmann@laborberlin.com; 9 Xiamen Innovax Biotech Co., Ltd., Haicang, Xiamen 361022, China; jwshih@innovax.cn; 10 Department of Gastroenterology, Hepatology and Endocrinology, Hannover Medical School, 30623 Hannover, Germany; Wedemeyer.heiner@mh-hannover.de; 11 German Center for Infection Research, Partner Hannover-Braunschweig, 38124 Braunschweig, Germany; 12 Faculty of Medicine, Duy Tan University, Da Nang 550000, Vietnam

**Keywords:** hepatitis E, infection, outbreak, epidemiology, global health

## Abstract

Infection with hepatitis E virus (HEV) represents the most common source of viral hepatitis globally. Although infecting over 20 million people annually in endemic regions, with major outbreaks described since the 1950s, hepatitis E remains an underestimated disease. This review gives a current view of the global circulation and epidemiology of this emerging virus. The history of HEV, from the first reported enteric non-A non-B hepatitis outbreaks, to the discovery of the viral agent and the molecular characterization of the different human pathogenic genotypes, is discussed. Furthermore, the current state of research regarding the virology of HEV is critically assessed, and the challenges towards prevention and diagnosis, as well as clinical risks of the disease described. Together, these points aim to underline the significant impact of hepatitis E on global health and the need for further in-depth research to better understand the pathophysiology and its role in the complex disease manifestations of HEV infection.

## 1. Introduction

Hepatitis E virus (HEV) is a quasi-enveloped, positive strand RNA virus belonging to the family *Hepeviridae* [1]. HEV is the causative agent of Hepatitis E, the most common cause of acute viral hepatitis both in resource poor and developed countries. Hepatitis E presents as a mostly asymptomatic or acute self-limiting disease with a mortality rate up to 3% in young adults [2]. However the mortality rate may reach 30% in pregnant women [3]. Furthermore, chronic hepatitis E infections may occur in high-risk groups such as immunocompromised individuals (e.g., transplant recipients), those with pre-existing liver disease, HIV-positive persons, and cancer patients [4,5,6,7]. A recent study estimates that 939 million people worldwide have been infected with HEV in the past and that 15–110 million people have recent or ongoing infections [8]. According to the WHO, an estimated 3.3 million symptomatic hepatitis E cases occur each year in endemic areas with 44,000 related deaths [9]. 

HEV belongs to the genus *Orthohepevirus*, containing four species, namely *Orthohepevirus A, B, C,* and *D* with *A* containing the genotypes HEV-1 to HEV-8 [10]. The genotypes that infect humans include HEV-1 to -4, -7, and *Orthohepevirus C*, casually termed rat HEV [10,11,12,13]. HEV-1 and -2 infect only humans and cause large waterborne outbreaks due to contaminated drinking water in endemic regions of South and Southeast Asia, Africa, and Mexico [14]. HEV-3 and -4 infect both humans and animals, cause sporadic cases in developed countries, and are mainly spread through consumption or close contact with contaminated animal products [14,15]. HEV-7 and rat HEV infections are rarely reported. HEV-1 is mainly transmitted via the fecal–oral route, but also by vertical transmission from mother to child, from person to person, and by blood transfusions [14,16]. HEV-2 is transmitted via the fecal–oral route and human-to-human [14]. In contrast, HEV-3 and -4 are transmitted by transfusion of contaminated blood products, consuming contaminated shellfish, contact with infected animals, environmental contamination by animal manure run-off, and consumption of raw or undercooked meat [14,16]. Hepatitis E is not a sole health burden of the developing world, however, with numbers of reported sporadic cases increasing in industrialized nations, where the virus is spread primarily through zoonotic transmission.

Although usually a self-limiting disease in immunocompetent persons, hepatitis E can cause serious complications in at-risk populations such as pregnant women [17] and organ transplant recipients [5,18,19]. Treatment options remain limited, and only one vaccine has been developed so far with its use limited to China [20].

This review focuses on the history and discovery of the hepatitis E virus and discusses reported outbreaks in order to share new aspects and insights into its epidemiology and global circulation. Furthermore, it explores studies on the virology of HEV to portray a detailed picture of the known stages of the viral life cycle. Finally, it briefly summarizes the clinical aspects to underline the significant need for further research into the viral pathophysiology contributing to this underrated emerging infectious disease.

## 2. Discovery and History

An epidemic of HEV was reported in 1955 in Delhi, India, with about 29,000 cases of icteric hepatitis [21]. After this epidemic, several waterborne outbreaks were reported throughout India, and most of these cases were non-A and non-B, leading the disease to be described as enteric non-A non-B hepatitis (ENANBH) [21]. In addition, a major water-related epidemic outbreak in the Kashmir Valley was reported at the end of 1978, with 52,000 cases and 1700 deaths [22,23]. The symptoms of these cases were similar to hepatitis A but were negative for both hepatitis A and hepatitis B and were therefore confirmed as ENANBH [22]. In 1981, hepatitis occurred in a Soviet military camp in Afghanistan. To investigate this situation, a doctor in the Russian army, Mikhail Balayan, voluntarily ingested a pooled filtrate of stool samples from the infected soldiers, and he subsequently developed acute hepatitis [24]. The serum of Dr. Balayan was negative for hepatitis A virus (HAV) and hepatitis B virus (HBV), suggesting that a new pathogen was responsible for this infection. Immunoelectron microscopy identified 20–30 nm non-enveloped virus-like particles in stool, confirming a novel ENANBH virus [24]. In 1990, this novel ENANBH was partially cloned and sequenced and was henceforth called the hepatitis E virus (HEV) [25,26]. It was initially indicated that hepatitis E infection spreads via contaminated water and is limited to resource poor countries. Later, however, increasing reports of sporadic cases emerged in non-endemic industrialized countries with high seroprevalence in a few areas of the United States [27]. The reason for this high prevalence was not understood, and it was speculated that undetected non-pathogenic or less pathogenic HEV strains were circulating. In 1998, genome sequences confirmed that human HEV were similar to that of HEV in pigs, suggesting zoonotic transmission pathways [28,29]. Overall, these studies led to the identification of a broad spectrum of HEV strains that are either restricted to humans, animals, or infect both.

## 3. Virology

**Figure 1:**
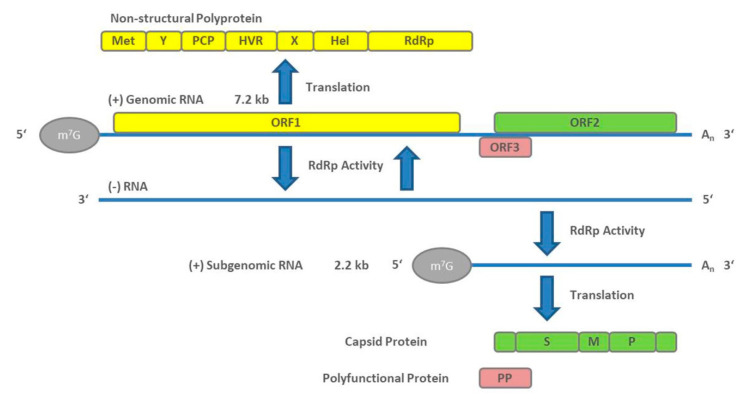
Genome arrangement of hepatitis E virus (HEV) and steps of viral genome replication.

HEV particles have an icosahedral shape, are non-enveloped, and form virions with a diameter of about 27–34 nm [30]. The HEV genome is about 7.2 kb in size and consists of a 5′ UTR capped with 7-methylguanosine (M^7^G), followed by three open reading frames (ORF1, ORF2, and ORF3). The 3′ UTR ends with a poly(A) tail (A_n_) like the eukaryotic mRNA structure. Viral replication starts with the translation of the ORF1-encoded non-structural polyprotein. An RNA-dependent RNA polymerase (RdRp) subsequently transcribes the full-length negative-sense RNA. This RNA serves as a template for the synthesis of two viral positive-sense RNAs in infected cells, a full-length genomic RNA, and a subgenomic RNA containing the capsid protein-encoding ORF2 and polyfunctional protein (PP)-encoding ORF3 [31]. The genomic arrangement and the stages of viral genome replication and protein synthesis are visualized in Figure 1.

ORF1 encodes a non-structural polyprotein of varying length that consists of seven domains comprising a methyl transferase (Met), X and Y domains, a papain-like cysteine protease (PCP), a proline-rich hypervariable region (HVR), RNA helicase (Hel), and RNA-dependent RNA polymerase (RdRp) [32]. Of these seven, only the Met, Hel, and RdRp have been functionally well characterized [33,34,35,36]. The complete function of all ORF1 domains is still not fully understood. In addition, whether it functions as a multi-domain polyprotein like a “Swiss army knife” or whether it needs to be cleaved for functional activity [37] is under debate and there is still no conclusive evidence that PCP has a protease function [38]. Although ORF1 is essential for HEV replication, its HVR displays considerable sequence divergence even between isolates of the same virus genotype [39]. Size differences between different HEV genomes can be primarily attributed to the HVR region. Analysis of different patient derived HEV isolates revealed various strains that harbor insertions from other regions of the viral genome, or from human genes, within the HVR [40].

The ORF2 encodes for the capsid protein. Its N-terminal signal peptide shuttles it to the extracellular compartment. The ORF2 protein contains three potential *N*-glycosylation sites [41,42,43], and is the main immunogenic target of neutralizing antibodies [41,44]. The full-length ORF2 encodes 660aa; however, recent reports suggested that ORF2 protein is processed into at least two forms, including one or two forms of secreted glycoproteins that are not associated with infectious particles, and one unglycosylated form which is the structural component of infectious particles [45,46]. ORF2 has been well characterized for its usefulness in diagnostics and vaccines, including the vaccine Hecolin^®^ p239, which is currently only approved in China.

The ORF3 protein is a polyfunctional 13-kDa protein of 113 (genotype 3) or 114aa (genotypes 1, 2, and 4). Computational homology scans did not reveal any domains comparable to other known viral proteins. It has been shown to bind to microtubules and be involved in particle assembly and egress by interaction with the tumor susceptibility gene 101 protein (TSG101), a key protein involved in the endosomal sorting complexes of the ESCRT (endosomal sorting complexes required to transport) transport pathway and involved in the budding of the viruses [47,48,49,50]. Furthermore, it may play a role in infectious particle secretion via its palmitoylation and membrane association [51]. Additionally, there are also reports of its role in intracellular transduction pathways, the potential to reduce host immune responses, and protection of virus-infected cells [52,53,54]. A recent article reports that ORF3 is a functional ion channel required for release of infectious particles [55]. 

HEV exists as quasi-enveloped viral particles in blood and cell culture supernatant and as non-enveloped virions in bile and feces [56]. When they are shed into the environment, non-enveloped naked virions are enterically transmitted through contaminated water or food. So far, it is not well understood how HEV virions overcome the intestinal barrier and reach the liver. However, it is assumed that the virions first infect the enterocytes, where they multiply, and are excreted as quasi-enveloped virus particles into blood circulation, thus infecting hepatocytes [57]. On their way through the bile duct, the envelope is stripped off and naked, and more infectious virions are again released via the stool [56,58].

Naked HEV particles possibly attach to target cells via heparin sulfate proteoglycans (HSGPs) [59] and heat shock cognate protein 70 (HSC70) [60]. Integrin α3 has been described recently [61] as a candidate receptor to mediate entry into the cells by dynamin-dependent, clathrin-mediated endocytosis, supported by the GTPases Ras-related proteins Rab5A (RAB5) and Rab7a (RAB7), which are necessary for quasi-enveloped particle internalization [59,62,63]. Quasi-enveloped particles attach less efficiently to cells and likely enter the cell in a manner similar to exosomes [63]. Not requiring HSGPs allows attachment in a non-cell-specific manner, possibly explaining HEV’s capacity to infect extrahepatic tissues [63]. Following this, lysosomal degradation of the lipid membrane (in the case of enveloped particles) and subsequent viral capsid uncoating take place, followed by release of the genomic HEV positive strand RNA into the cytoplasm [63]. The host cellular transcriptional machinery translates ORF1 polyprotein containing RdRp from HEV RNA. The polymerase transcribes the full-length negative-sense HEV RNA. From this negative strand, two RNAs are transcribed by RNA helicases and RdRp to form a full-length genomic RNA and a 2.2 kb bicistronic subgenomic RNA. These two capped and polyadenylated RNAs serve as templates for the translation of non-structural ORF1 polyproteins, ORF2 capsid proteins, and polyfunctional ORF3 proteins [64].

The subsequent steps are viral assembly and release. ORF2 and ORF3 and positive-sense genomic HEV RNA are known to form a complex in the ER–Golgi intermediate compartment and produce viral progeny particles [65,66]. The particles of the progeny virus bind to the TSG101 protein and are secreted in a presumably basal fashion as enveloped particles [47,48]. When leaving hepatocytes from the apical part, the envelope is stripped as described above [56,58]. There are many significant gaps in the understanding of the HEV life cycle and virus–host cell interactions [67], and further studies are urgently needed. Several in vitro systems exist to study the virus. Reverse genetics models based on infectious cDNA clones have been described for several genotypes. The most commonly used are the Sar-55-related genotype 1 clone [68], the genotype 3a and 3c Kernow-C1- [69], and 47832-related [70] clones, respectively, both of which contain insertions in the HVR, and the genotype 4 TW6196 clone [71]. A recent presentation of a novel in vitro method to produce high viral titers, allowing study of the full HEV replication cycle in cell culture, has additionally created confidence that we may overcome our limited understanding of HEV pathophysiology [72]. Although these systems are most commonly used in conventional cell culture systems, several animal infection models have been developed. Rhesus monkeys have been shown to be susceptible to HEV-1 through -4 [28,73], while cynomolgous monkeys and chimpanzees have been used as models for HEV-1 and -2 [68,74]. As natural hosts for HEV-3 and -4, pigs can be readily infected by strains of these genotypes [75,76]. Recent reports have also described successful infection of human liver chimeric mice with HEV-1 and -3 strains [77,78]. Moreover, small animal and avian models exist for the study of animal HEV. Although animal infection models provide more physiological conditions than cell culture systems, limitations are also present. Neither non-human primates nor mice represent natural hosts of HEV, while pigs can only be infected with HEV-3 and -4. Experiments with primates also raise ethical concerns.

## 4. Outbreaks

HEV outbreaks occur mainly in resource poor countries, due to waterborne infections caused by HEV-1 and possibly HEV-2 [79], often during the monsoon season. The outbreaks are caused mainly by consumption and use of contaminated water where sanitary and hygienic conditions are poor [14]. The first laboratory confirmed HEV outbreak was reported in Delhi in 1955, and since then several outbreaks have been reported in tropical and subtropical regions of the world, especially in Asian and African countries. Reported outbreaks are summarized in Table 1. 

The largest outbreaks involved 79,000 ENANBH cases in Kanpur, India, between 1990 and 1991, and 119,000 cases in China between 1986 and 1988 [93,94,95]. Sporadic outbreaks have also occurred in recent years in Asian and African countries, but with a low number of reported cases [133,134,135,136,137]. Using modelling approaches, a recent study describes the ecologically most suitable hotspots for HEV viruses: the Ganges Valley in India and Pakistan [138]. Important factors contributing to water-related outbreaks of HEV are population density, socio-economic conditions, the level of sanitation, and access to drinking water [14,138]. There are also frequent outbreaks in refugee camps, military camps, and emergency shelters in conflict- and catastrophe-affected regions [127,139,140,141,142]. In summary, HEV outbreaks can be prevented by improving sanitary conditions and ensuring access to clean drinking water.

## 5. Epidemiology

Taxonomically, the family *Hepeviridae* is divided into the genus *Orthohepevirus* with the species *Orthohepevirus A, B, C*, and *D*, and the genus *Piscihepevirus*. The species *Orthohepevirus A* contains eight distinct genotypes that infect humans and other mammals [10]. It is known that HEV-1 to -4 infect humans, and rare cases of human infection with other genotypes like HEV-7 have also been described [11,143]. Details on the genotypes of *Orthohepevirus A* including host range, transmission routes, and global distribution are summarized in Table 2.

**Figure 2:**
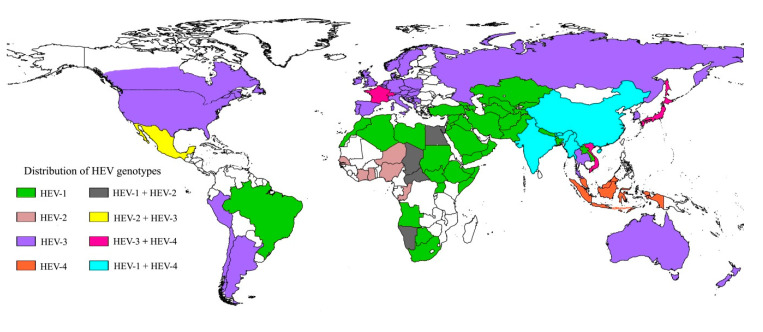
Global HEV genotype distribution. Different colors on the map indicate the distribution of HEV genotypes (HEV-1 through -4) across the globe. The figure was created using SimpleMappr, an online tool to produce publication-quality point maps [149].

Moreover, sporadic human infections with rat HEV *(Orthohepevirus C*) have been reported [12,13,144]. HEV epidemiology shows two differing patterns based on the global distribution of the *Orthohepevirus A* genotypes [9] (Figure 2). HEV-1 and HEV-2 genotypes are human pathogens that are mostly transmitted via the fecal–oral route in resource poor countries, mainly through contaminated drinking water, with several cases of vertical transmission having also been described [16]. In contrast, infections with HEV-3 and HEV-4 are autochthonous in developed countries and mainly zoonotically acquired via consumption of contaminated food, but parenteral transmission via blood transfusions have also been described [145]. Despite several HEV genotypes, only a single serotype has been reported, which simplifies seroprevalence studies, diagnosis, and vaccination [146,147,148].

The HEV-1 and HEV-2 genotypes are estimated to infect about 20 million people in resource poor countries, resulting in 3.3 million symptomatic cases and 44,000 deaths per year [9]. However, it is believed that these figures are underestimated due to lack of awareness and lack of diagnosis and serological tests [150]. HEV-1 is the main cause of significant recurrent epidemics in resource poor countries in Africa and Asia and isolated small epidemics in Latin America, as well as large outbreaks in India [14,151]. HEV-2 outbreaks were reported in 1986–1987 from rural towns in Morelos in Mexico [152], Nigeria, Sudan, and Namibia [79,153,154,155], and the recent ones reported in Burkina Faso. Studies have shown that HEV-1 and HEV-2 infections occur most frequently in men during their adolescence (15–30 years), and most patients experience acute self-limiting hepatitis [94,100]. In addition, HEV outbreaks were recognized among people living in refugee camps and in emergency shelters after natural disasters [127,139,140,141,142]. Few studies have reported human-to-human transmission; however, this needs to be elucidated [156].

HEV-3 and HEV-4 are zoonotically transmitted from pigs, wild boar, deer, and rabbits in developed countries [157], where pigs are the main reservoir, and autochthonous infections are caused by the consumption of undercooked meat [158] or contact with pigs and wild animals [159]. Previously, the cause of HEV-induced hepatitis was considered a disease of resource poor countries, but it has become clear that HEV is also acquired locally in industrialized regions [160,161]. A recent review and meta-analysis lists seroprevalence rates of 0.04% to 52.5% in these countries, with the highest prevalence reported from France, Poland, and Denmark [162]. HEV is much more frequent than previously thought, with an estimated >400.000 infections per year in Germany [163]. HEV-3 is common among pigs worldwide, which constitute the major reservoir for human infections [164,165]. HEV-4 is widely distributed in South and East Asia, where both wild and domesticated pigs serve as a reservoir [10,158,166]. Infection rates are high in people over 50 years of age [4,167,168], and seroepidemiological studies found no significant differences in HEV-IgG seropositivity according to gender [169]. However, a recent study revealed that men were more frequently infected than women [170]. Other HEV genotypes such as HEV-5 and -6 were found in wild boars, whereas HEV-7 and -8 are present in camels [143,171,172,173]. So far, only HEV-7 has shown a zoonotic potential associated with practices of camel meat consumption in the Middle East [11]. Sporadic reports of human infections with rat HEV (*Orthohepevirus C*) have also been reported [12,13]. HEV genotypes are selective with unique transmission patterns, pathogenesis [174,175], clinical manifestations in different patient groups, and host adaption.

## 6. Diagnosis and Clinical Aspects 

As a large percentage of HEV infections do not present with symptoms, the disease often goes undiagnosed. In individuals presenting with hepatitis, symptoms are indistinguishable from those of hepatitis A. Standard diagnostic tests for hepatitis E include the detection of HEV-specific antibodies, including both anti-HEV IgM and IgG, as well as amplification of the viral genome using conventional PCR or real-time reverse transcription PCR [176]. During the last decades, WHO international standard materials have been developed to assist standardization of NAT and anti-HEV IgG detection assays [177,178]. Awareness of the need for HEV testing has been increasing over the last decade, and many countries have begun to implement HEV RNA screening in blood donations [179].

Although the main site of replication for HEV is the liver, non-hepatic replication has been shown in the brain, kidney, placenta, and others [180]. This may explain a series of different extrahepatic manifestations that can occur in infected patients. Neurological manifestations include Guillain–Barré syndrome and neuralgic amyotrophy, while kidney disorders such as cryoglobulinemia and hematological conditions have also been described in association with hepatitis E [181,182,183,184,185,186,187]. In addition, deaths due to fulminant hepatitis have been reported in 0.5 to 4% of patients, and this percentage is known to be higher in people with pre-existing liver disease [188] and among young children [189]. HEV superinfection could additionally influence the outcome and progression of HBV-related liver diseases [190]. Furthermore, severe disease outcomes may occur in pregnant women infected with HEV-1 with studies describing maternal mortality rates >30% within the third trimester, mainly due to fulminant hepatic failure [17]. In immunocompromised persons such as solid organ and hematopoietic stem cell transplant recipients, HEV infection may persist. Patients with chronic hepatitis E will often rapidly progress to liver cirrhosis and have an increased mortality rate [158]. There is no approved drug against HEV infection. If initial reduction of immunosuppression does not lead to HEV clearance, off-label treatment for chronic hepatitis E is indicated with ribavirin. Non-responding liver-transplant patients can be further treated with pegylated interferon alfa-2a and pegylated interferon alfa-2b [168]. Several molecules with in vitro antiviral activity against HEV have been described. Repurposed antivirals include 2′-C-methylcytidine (2-CMC) [191], NITD008 and GPC-N114 [192], ciprofloxacin and IFN-λ [193], and sofosbuvir [194]. Compound screening studies have revealed plant ethanol extracts from *L. mauritiana* [195] and *L. platyphylla* [196], as well as zinc [197], and the small molecule 66E2 [198] as potential antivirals. An FDA-approved drug, deptropine, a histamine H1 receptor antagonist used in asthma treatment, showed anti-HEV activity in cell culture and synergized with ribavirin [199]. Target-guided approaches include peptide conjugated phosphorodiamidate morpholino oligomers (PPMO) [200] and proteasome inhibitor MG132 [201,202] in HEV-1 cell culture, the cyclic peptide CP11, which inhibits interaction of HEV with the ESCRT [203], several inhibitors of nucleotide synthesis, including mycophenolic acid [204], Brequinar, and Leflunomide [205], and the natural compound silvestrol [206]. However, only silvestrol [206] and the hepatitis C virus polymerase inhibitor sofosbuvir [194] advanced to in vivo studies. Unfortunately, sofosbuvir monotherapy failed to prove its potency to reduce HEV viral loads in a phase II pilot trial [207]. Finally, the only developed vaccine against HEV, Hecolin^®^, is thus far only approved in China [20]. However, several other candidates based on the HEV capsid protein are currently being developed using different expression systems, such as baculovirus-infected insect cells [208], prokaryotic systems [209], expression in yeast [210,211], vectored vaccines [212], and chimeric vaccines [213,214]. So far, apart from Hecolin^®^, only two candidates have entered clinical trials [215,216]. An effective vaccine is an important step in preventing severe hepatitis E-related liver disease in risk populations such as pregnant women and immunocompromised patients.

## 7. Conclusions

Infection with hepatitis E virus is still an under-reported disease worldwide. However, awareness of the prevalence of this virus has grown exponentially in the last decades. Before and since its discovery, there had been numerous large-scale outbreaks in the developing world with tens of thousands of cases, demonstrating that HEV is the most common cause of viral hepatitis worldwide. While HEV-1 and HEV-2 occur mainly in waterborne epidemics in areas with insufficient hygienic conditions, sporadic cases of HEV-3 and HEV-4, which are transmitted zoonotically or via contaminated blood products in industrialized countries, have confirmed that HEV is not a health burden solely of resource poor regions. Although much effort has been put into elucidating the mechanisms of infection, replication, and pathology of the virus, many questions are still left unanswered, underlining the importance of further research on its virology. This is especially important as hepatitis E infections—depending on the genotype—can cause severe liver disease in risk groups such as pregnant women and immunocompromised persons. With these aspects in mind, it is clear that further research is vital to develop more effective treatment options and approved vaccination strategies.

## Figures and Tables

**Table 1:** Reported HEV outbreaks.

Year(s)	Country	Mode of Transmission	Reported	Ref.
1955–1956	India	Waterborne	29,300	[80]
1978–1979	Kashmir	Waterborne	>270	[23]
1980–1981	Algeria	Sewage contamination—river water	788	[81]
1982	Myanmar	Waterborne	399	[82]
1983	Namibia	Waterborne	hundreds	[79,83]
1983–1984	Cote d’Ivoire	Waterborne	623	[84]
1985	Turkmenistan	Waterborne	16,175	[85,86]
1985	Botswana	Fecal contamination of water	273	[87]
1986	Mexico	Contaminated well water	>200	[88]
1988	Somalia	Waterborne	106	[89]
1988–1989	India	Contaminated drinking water	53	[90]
1988–1989	Ethiopia	After monsoon rains	>750	[91]
1989	Myanmar	Contamination—water supply by feces	93	[92]
1991	India	Contaminated river water (Ganges)	79,000 ^1^	[93]
1991	China	Waterborne	119,000	[94,95]
1991	Indonesia	Waterborne	1688 ^1^	[96]
1993–1994	Pakistan	Waterborne	3827	[97]
1994	Vietnam	After heavy rains	>300	[98]
1994	Morocco	Fecal contamination of drinking water	73	[99]
1995–1996	Namibia	Waterborne	>600	[79]
1998	India	Waterborne	82	[100]
1998	Indonesia	Waterborne	472	[101]
1998	Pakistan	Fecal contamination—water system	104	[102]
2002	India	Contaminated water	185	[103]
2004	Central African Republic	Rainy season	213	[104]
2004	Sudan	Safe water insufficient	>2600	[105]
2004	India	Drinking untreated raw river water	538	[106]
2004	Chad	Waterborne	1442 ^1^	[107]
2005	Iraq	Waterborne	268 ^1^	[108]
2005	India	Contaminated drinking water	429	[109]
2006	Sudan	Waterborne	2621	[110,111]
2007–2008	India	Fecal contamination of water resources	64	[112]
2007–2008	Egypt	Waterborne	28	[113]
2007–2009	Uganda	Waterborne	146	[114]
2008	India	Sewage contamination of the river	23,915 ^1^	[115]
2008	Uganda	Substantial person-to-person	>10,000	[116,117,118]
2008–2009	Bangladesh	Sewage contamination—municipal water	4751 ^1^	[119]
2009–2012	Uganda	Contaminated water	987	[120]
2010	Bangladesh	Waterborne	200	[121]
2010	India	Waterborne	102	[122]
2005–2010	India	Waterborne	442	[123]
2010–2011	Sudan	Waterborne	39	[105]
2012	India	Fecal contamination of drinking water	180	[124]
2012	Kenya	Waterborne	131	[125]
2008–2012	Central African Republic	Waterborne	745	[126]
2012–2013	South Sudan	Waterborne	5080 ^1^	[127]
2013	India	Sewage contamination of drinking water	240	[128]
2013	Cameroon	Waterborne	33	[129]
2014–2015	Bangladesh	Waterborne	103	[130]
2014–2016	India	Waterborne	17	[131]
2016–2017	Chad	Waterborne	1293	[132]
2017–2018	Nigeria	Contamination of drinking water	1376	[133]
2014–2017	Bangladesh	Waterborne	661	[134]
2018	Central African Republic	Waterborne	149	[135]
2018	South Sudan	Waterborne	161	[135]
2019	Pakistan	Waterborne	300	[136]
2017–2020	Namibia	Waterborne	7247	[137]

^1^ Numbers based on reported estimates.

**Table 2:** Details on *Orthohepevirus A* genotypes.

Genotype ^1^ (Subtypes)	Host	Transmission Route	Global Distribution ^1^
HEV-1 (1a–1g)	Human, primates	Fecal–oral via contaminated drinking water	Mainly resource poor regions in India, Pakistan, Bangladesh, Myanmar, China, Mongolia, Morocco, Chad, Nigeria
HEV-2 (2a, 2b)	Human, primates	Fecal–oral via contaminated drinking water	Mainly resource poor regions in Mexico, Nigeria
HEV-3 (3a–3m, 3ra)	Human, pig, wild boar, deer, mongoose, rabbit (3ra), hare (3ra), rodents	Zoonotic via consumption or contact of/with contaminated foodstuffs; parenteral via contaminated blood donations	Mainly in industrialized countries such as USA, Canada, China, Japan, South Korea, India, Singapore, UK, Germany, France, Italy, Spain, Sweden, Switzerland, Netherlands, Denmark, Hungary
HEV-4 (4a–4i)	Human, pig, wild boar, cow, goat, yak, Rhesus monkey	Zoonotic via consumption or contact of/with contaminated foodstuffs; parenteral via contaminated blood donations	Mainly in Asian countries such as China, Mongolia, Japan, South Korea, Taiwan, Cambodia, India
HEV-5 (5a)	Wild boar	Unavailable	Japan
HEV-6 (6a)	Wild boar	Unavailable	Japan
HEV-7 (7a)	Human, dromedary camel	Zoonotic, likely via consumption of camel meat	UAE
HEV-8 (8a)	Bactrian camel	Unavailable	China

^1^ Information according to the International Committee on Taxonomy of Viruses (ICTV) as of September 2020 and Smith et al. 2020 [10].
